# Gut Metagenome as a Potential Diagnostic and Predictive Biomarker in Slow Transit Constipation

**DOI:** 10.3389/fmed.2021.777961

**Published:** 2022-02-08

**Authors:** Hongliang Tian, Chen Ye, Bo Yang, Jiaqu Cui, Zhijun Zheng, Chunyan Wu, Shailan Zhou, Xiaoqiong Lv, Nan Qin, Huanlong Qin, Ning Li, Qiyi Chen

**Affiliations:** ^1^Intestinal Microenvironment Treatment Center of General Surgery, Tenth People's Hospital of Tongji University, Shanghai, China; ^2^Clinical Research Center for Digestive Diseases of Tongji University, Shanghai, China; ^3^Realbio Genomics Institute, Shanghai, China

**Keywords:** slow transit constipation, gut microbiome, metagenomic analysis, pathogenesis, diagnostic, biomarker

## Abstract

Slow transit constipation (STC) is one of the most frequent gastrointestinal diagnoses. In this study, we conducted a quantitative metagenomics study in 118 Chinese individuals. These participants were divided into the discovery cohort of 50 patients with STC and 40 healthy controls as well as a validation cohort of 16 patients and 12 healthy controls. We found that the intestinal microbiome of patients with STC was significantly different from that of healthy individuals at the phylum, genus, and species level. Patients with STC had markedly higher levels of Alistipes and Eubacterium and lower abundance of multiple species belonging to the *Roseburia* genus. Patients with STC gene expression levels and the Kyoto Encyclopedia of Genes and Genomes (KEGG) orthology pathway (such as fatty acid biosynthesis, butanoate metabolism, and methane metabolism pathways) enrichment were also substantially different from those of healthy controls. These microbiome and metabolite differences may be valuable biomarkers for STC. Our findings suggest that alteration of the microbiome may lead to constipation by changing the levels of microbial-derived metabolites in the gut. Above findings may help us in the development of microbial drugs.

## Introduction

Chronic constipation is characterized by various symptoms such as straining, lumpy or hard stool, sensation of incomplete evacuation, sensation of anorectal obstruction, and infrequent defecation (<3 defecations/week) (1, 2). Slow transit constipation (STC) is the major category of chronic constipation (3). The characteristics of STC include slower colonic transit and fewer high-amplitude propagated contractions. It is a complex pathogenesis that involves the enteric nervous system, the interstitial cells of Cajal, and colonic smooth muscle, but it is still incompletely understood (4–7).

Decreased colonic motility is an important pathophysiological mechanism of STC. Recently, several studies have suggested that gut microbiota may be involved in the etiology of constipation. Disturbance of stool microbiota has been found in many patients with constipation (8, 9). Furthermore, Vandeputte et al. found that stool consistency is associated with the richness and composition of gut microbiota as well as enterotypes and bacterial growth rates (10). Recent advances in sequencing technology have profoundly affected the field of microbiology. Quantitative metagenomics analysis has been used successfully to study the pathogenesis of many chronic diseases (11, 12). However, to date, no study has used this approach to analyze STC.

In this study, to comprehensively catalog the gut microbiome features of constipation, we conducted a quantitative metagenomics study in 118 adults, including STC and healthy controls.

## Methods

### Study Participants

A total of 66 patients with STC and 52 healthy controls from September 2015 to December 2019 were enrolled in this study from the Shanghai Tenth People's Hospital. Inclusion criteria: Ages eligible for study: 18 years and older; body mass index: 18–24 kg/m^2^; and STC as defined by patients with a colonic transit test (CTT) of >48 h (13, 14). This clinical trial was registered at ClinicalTrials.gov (NCT02395484). The clinical diagnosis and fecal samples of all the individuals were obtained from the hospitals (Table 1).

**Table 1:** Characteristics of patients with slow transit constipation (STC) and healthy controls.

	**STC patients** **(*n* = 66)**	**Healthy controls** **(*n* = 52)**
Gender (M:F)	14:26	17:23
Age (y)	43.2 ± 2.6	45.2 ± 3.5
Weight (kg)	58.2 ± 3.2	59.7 ± 6.3
Height (cm)	166 ± 5.2	164 ± 7.2
BMI	20.4 ± 2.1	22.4 ± 1.6
Stools/day (week)	1.6 ± 0.8	5.4 ± 1.1
Stool consistency (1–7)	2.1 ± 0.8	4.2 ± 0.6
Medications history	Dietary modification, laxatives (including osmotic and stimulant laxatives), and biofeedback	None
Disease history (years)	6.1 ± 3.8	None
Colonic transit test (hours)	83.4 ± 12.6	None

### Sample Collection and DNA Extraction

Fecal samples were collected from the recruited subjects from these 118 participants. All the samples were then frozen immediately and stored at −80°C until DNA extraction. DNA was extracted from each stool sample using the commercial QIAamp DNA Stool Mini Kit (Qiagen, Valencia, California, USA).

### Deoxyribonucleic Acid Library Construction and Sequencing

The metagenomic DNA libraries were constructed with 2 μg genome DNA according to the instruction of the manufacturer (Illumina, California, USA), with an average of 350 bp insert size. The quality of all the libraries was evaluated using the Agilent 2100 Bioanalyzer with a DNA 1000 LabChip Kit.

### Illumina Hiseq Sequencing

The Illumina Hiseq platform was employed to sequence the 118 samples, thereby obtaining a total of 1.6 Tbp sequencing data. Illumina raw reads were subjected to the following treatments: (1) reads with more than 3 ambiguous N bases were removed; (2) reads with less than 60% of high quality bases (Phred score ≥ 20) were deleted; and (3) 3′ end of reads were trimmed to the first high quality base. The subsequent high quality reads were further mapped to human genome by SOAPaligner (version 2.21) and any hit associated with the reads and their mated reads was removed. After QC, trimming, and remove host contaminate, 44.76 ± 0.86 million clean reads per sample on average are reserved. Sample statistic information is given in Supplementary Table S1.

### *De novo* Assembly of the Illumina Short Reads

SOAPdenovo (version 2.04), which is based on De Bruijn graph construction, was employed to assemble short reads with parameters “-M 3 -u -L 100 -d 1 -F.” k-mers, varying from 39 to 59 by 4, was tested for each sample (15). The resulting scaffolds were cut into contigs at ambiguous Ns and only contigs longer than 500 bp were saved. N50 was calculated for contigs of different k-mers and only the contigs of largest N50 assembly were attributed to a sample. All these contigs were applied for gene prediction by Meta-Gene Marker (version 3.25).

### Taxonomic and Gene Profiling

Microbial composition at each taxonomic level was calculated using the MetaPhlAn2 program with default parameters. The program is available at https://bitbucket.org/biobakery/biobakery/wiki/humann2. Relative abundances of the genes were calculated with the procedure introduced in Qin et al. (12). When calculating the abundance of genes, the high quality reads from each sample were aligned against the gene catalog by using SOAPalign2.21 with parameters of “-r 2 -m 100 - × 1,000” and only the both paired-end reads, which could be mapped to a same gene, were accepted.

### Gene Catalog, the Kyoto Encyclopedia of Genes and Genomes Database Annotation, and Pathway Profile

After removing redundancy by Collect DNA-HIT, genes were also annotated by the KEGG database. The KEGG Orthology (KO) profiling was calculated as the sum abundances of genes with the same KO number. The abundances of the KEGG pathway were calculated as the average abundances of all the KO under the pathway (16).

### Statistical Methods

Characteristics of participant between two groups were compared by using the *t*-tests for continuous variables and the chi-squared tests for categorical variables. The richness and the β-diversity of the microbiota dataset were analyzed using Quantitative Insights Into Microbial Ecology (17). The nonparametric Wilcoxon signed-rank test was employed to analyze the statistical significance of the gene, the KO, Ortholog Groups, enzyme, and different taxonomic (phylum, genus, and species) levels between the STC and healthy control group. The relative abundance of these features was subjected to statistical analyses. For categorical metadata and enterotype comparisons, samples were pooled into bins and significant features were identified using the Fisher's exact test with multiple testing correction of *p*-values. Statistical analyses were performed using SAS software (version 9.4; SAS Institute, Cary, North Carolina, USA) and R software (version 3.6.3; R Foundation for Statistical Computing, Vienna, Austria) (18).

## Results

### Enrollment of the Patient

Phenotype information was obtained from patients with STC and healthy controls during the discovery stage (90 samples) and the validation stage (28 samples), as shown in Table 1. The data obtained from these 118 samples are shown in Supplementary Table S1.

### Difference in Gut Microbiota Composition

**Figure 1:**
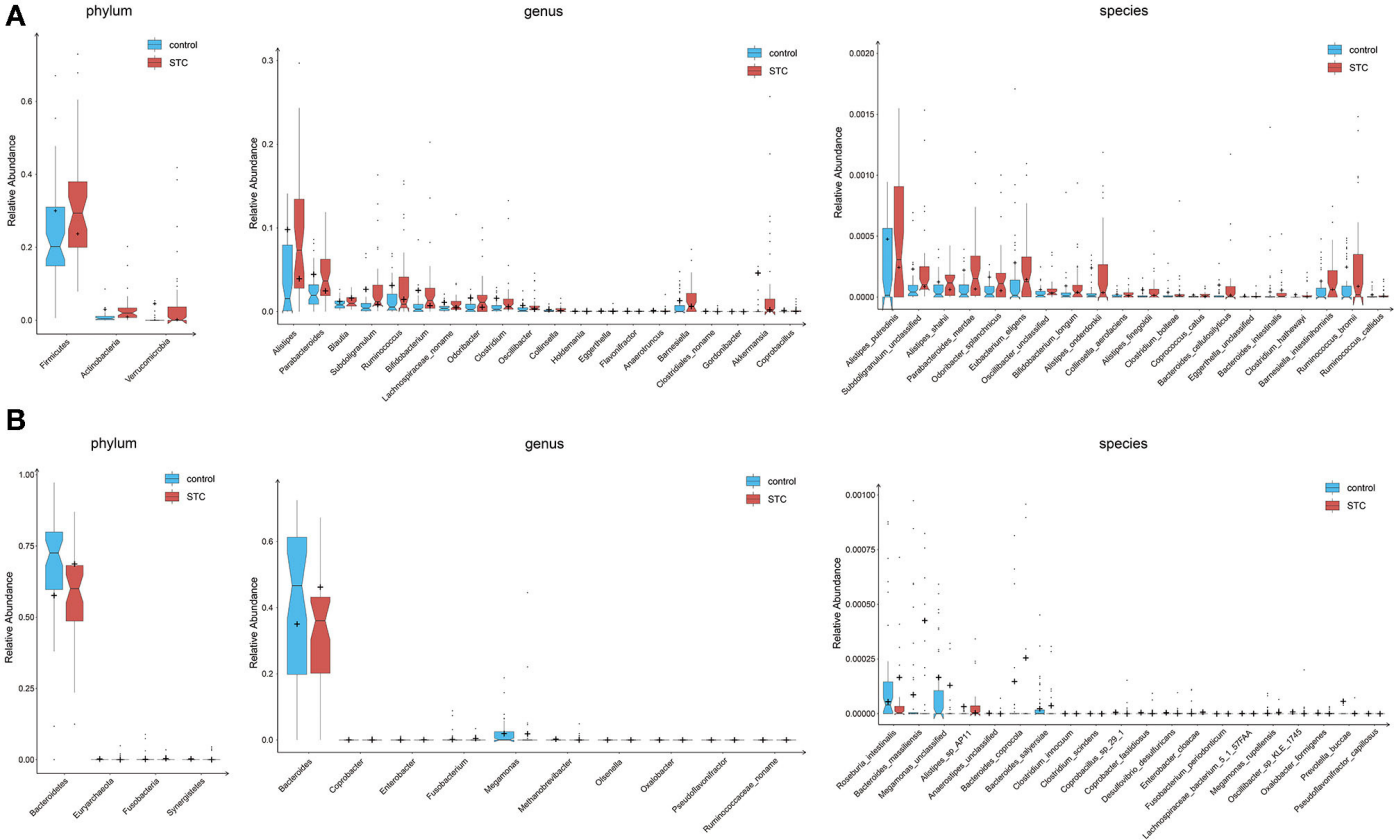
Differences of phylogenetic abundance between patients with slow transit constipation (STC) and healthy controls. The phylotypes were increased **(A)** or decreased **(B)** in patients with STC at the phylum, genus, and species levels. Red and blue indicate patients with STC and healthy controls, respectively. The phylogenetic abundance of phyla that had mean values <1% and that of genera and species that were <0.01% was excluded. After exclusion, the Wilcoxon rank-sum tests were applied to identify the differentially abundant phyla, genera, and species. Among these, the highest medians of the phylogenetic abundance in the enriched cohort were drawn as boxplots.

Our analysis revealed that STC displayed significant differences between two groups (Figure 1). At the phylum level, the intestinal flora in patients with STC was enriched in Firmicutes, Actinobacteria, and Verrucomicrobia and in healthy individuals was enriched in Bacteroidetes, Euryarchaeota, Fusobacteria, and Synergistetes (Supplementary Table S2). The genera enriched in patients with STC included Alistipes, Parabacteroides, Subdoligranulum, and Ruminococcus and the genera enriched in healthy individuals were Bacteroides, Roseburia, Haemophilus, and Klebsiella (Supplementary Table S3). At the species level, patients with STC were enriched in Alistipes putredinis, Parabacteroides merdae, Odoribacter splanchnicus, and Eubacterium eligens and healthy individuals were enriched in Roseburia intestinalis, Haemophilus parainfluenzae, Megamonas unclassified, and Klebsiella pneumoniae (Supplementary Table S4). Therefore, the intestinal microbiome of patients with STC is significantly different from that of healthy individuals at the phylum, genus, and species level.

### Detection of Enterotypes in Patients With STC

**Figure 2:**
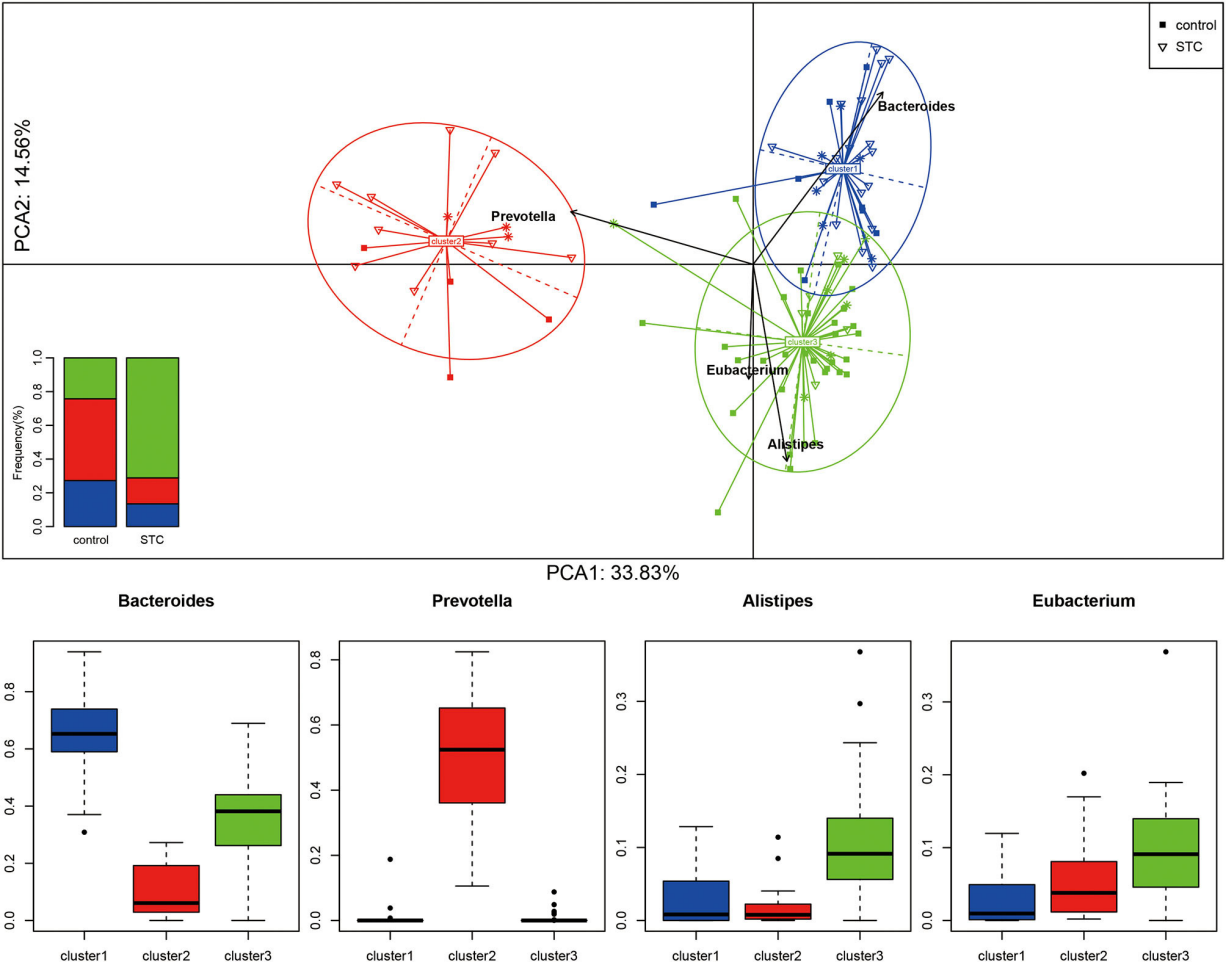
Sample clustering and classification for Enterotype analysis. According to the Jensen–Shannon distance of genus level, all the samples are clustered into the 3 types by the method of Manimozhiyan. Each of these 3 clusters are identifiable by the variation in the levels of 1 of 3 genera: Bacteroides (cluster 1), Prevotella (cluster 2), Alistipes and Ruminococcus (cluster 3). The histogram shows the distribution ratio of the control and STC groups in the 3 clusters.

Enterotype is a characteristic stratification of the intestinal microbiome population. To determine whether the patients with STC have a unique enterotype, we subjected 90 samples from cohort 1 to enterotype analysis, as described previously (19). The results showed that all the samples clustered into 1 of 3 enterotypes. The Bacteroides and Prevotella enterotypes were identical to those reported earlier (6) and the third enterotype was distinguished by high levels of Alistipes and Eubacterium (Figure 2; Supplementary Figure S1). The heatmap of top 30 most abundant genera for each of these 3 enterotypes is shown in Supplementary Figure S2.

### Differences in Microbial Gene and Pathway Expression Between Two Groups

The gut microbiota of patients with STC and healthy individuals included in this study differed greatly, not only at the phylum, genus, and species level, but also in terms of gene expression. Genes corresponding to the 859 KO entries were differentially expressed in these 2 groups: 609 genes were enriched in patients with STC and 250 genes were enriched in healthy controls (Supplementary Table S5). To determine the functional impact of these differences, we performed a pathway analysis and found that the most abundant KEGG orthologs in both the groups were those associated with fatty acid biosynthesis (Supplementary Figure S3). In the fatty acid biosynthesis pathway, 3-oxoacyl-(acyl-carrier-protein) synthase II [EC:2.3.1.179] (fabF, K09458), 3-hydroxyacyl-(acyl-carrier-protein) dehydratase [EC:4.2.1.59] (fabZ, K02372), enoyl-(acyl-carrier protein) reductase I [EC:1.3.1.9 1.3.1.10] (fabI, K00208), and long-chain acyl-CoA synthetase [EC:6.2.1.3] (K01897) were enriched in healthy controls, whereas acetyl-CoA carboxylase/biotin carboxylase 1 [EC:6.4.1.2] (K11262) and fatty acid synthase, bacteria type [EC:2.3.1] (K11533) were enriched in patients with STC (Supplementary Figure S4).

In the butanoate metabolism (map00650) pathway, succinate dehydrogenase/fumarate reductase, flavoprotein subunit [EC:1.3.5.1] (K00239) was enriched in healthy controls, while (R,R)-butanediol dehydrogenase/meso-butanediol dehydrogenase/diacetyl reductase [EC:1.1.1.4 1.1.1.-1.1.1.303] (K00004), succinate-semialdehyde dehydrogenase [EC:1.2.1.76] (K18119), acetate CoA/acetoacetate CoA-transferase alpha subunit [EC:2.8.3.8 2.8.3.9] (K01034), acetaldehyde dehydrogenase (acetylating) [EC:1.2.1.10] (K00132), 4-hydroxybutyrate dehydrogenase [EC:1.1.1.61] (K00043), 4-hydroxybutyrate CoA-transferase [EC:2.8.3.-] (K18122), 4-hydroxybutyryl-CoA dehydratase/vinylacetyl-CoA delta-isomerase [EC:4.2.1.120 5.3.3.3] (K14534), butyryl-CoA dehydrogenase [EC:1.3.8.1] (K00248), enoyl-CoA hydratase [EC:4.2.1.17] (K01692), glutaconate CoA-transferase, subunit A [EC:2.8.3.12] (K01039), 3-hydroxybutyryl-CoA dehydrogenase [EC:1.1.1.157] (K00074), acetyl-CoA C-acetyltransferase [EC:2.3.1.9] (K00626), and hydroxymethylglutaryl-CoA lyase [EC:4.1.3.4] (K01640) were enriched in patients with STC (Supplementary Figure S5).

In the methane metabolism (map00680) pathway, acetyl-CoA synthetase [EC:6.2.1.1] (K01895), phosphate acetyltransferase [EC:2.3.1.8] (K00625), malate dehydrogenase [EC:1.1.1.37] (K00024), and 2,3-bisphosphoglycerate-independent phosphoglycerate mutase [EC:5.4.2.12] (K15633) were enriched in healthy controls, whereas heterodisulfide reductase subunit A [EC:1.8.98.1] (K03388), 3-hexulose-6-phosphate synthase [EC:4.1.2.43] (K08093), (methyl-Co(III) methanol-specific corrinoid protein):coenzyme M methyltransferase [EC:2.1.1.246] (K14080), formylmethanofuran dehydrogenase subunit A [EC:1.2.7.12] (K00200), anaerobic carbon monoxide dehydrogenase, CODH/acetyl-CoA synthase (ACS) complex subunit alpha [EC:1.2.7.4] (K00192), acetyl-CoA decarbonylase/synthase, CODH/ACS complex subunit beta [EC:2.3.1.169] (K00193), 2-phosphosulfolactate phosphatase [EC:3.1.3.71] (K05979), and methanogen homoaconitase large subunit [EC:4.2.1.114] (K16792) were enriched in patients with STC (Supplementary Figure S6).

### Construction of a Model for Classifying Patients as Having STC Based on Intestinal Microbiome Biomarkers

**Figure 3:**
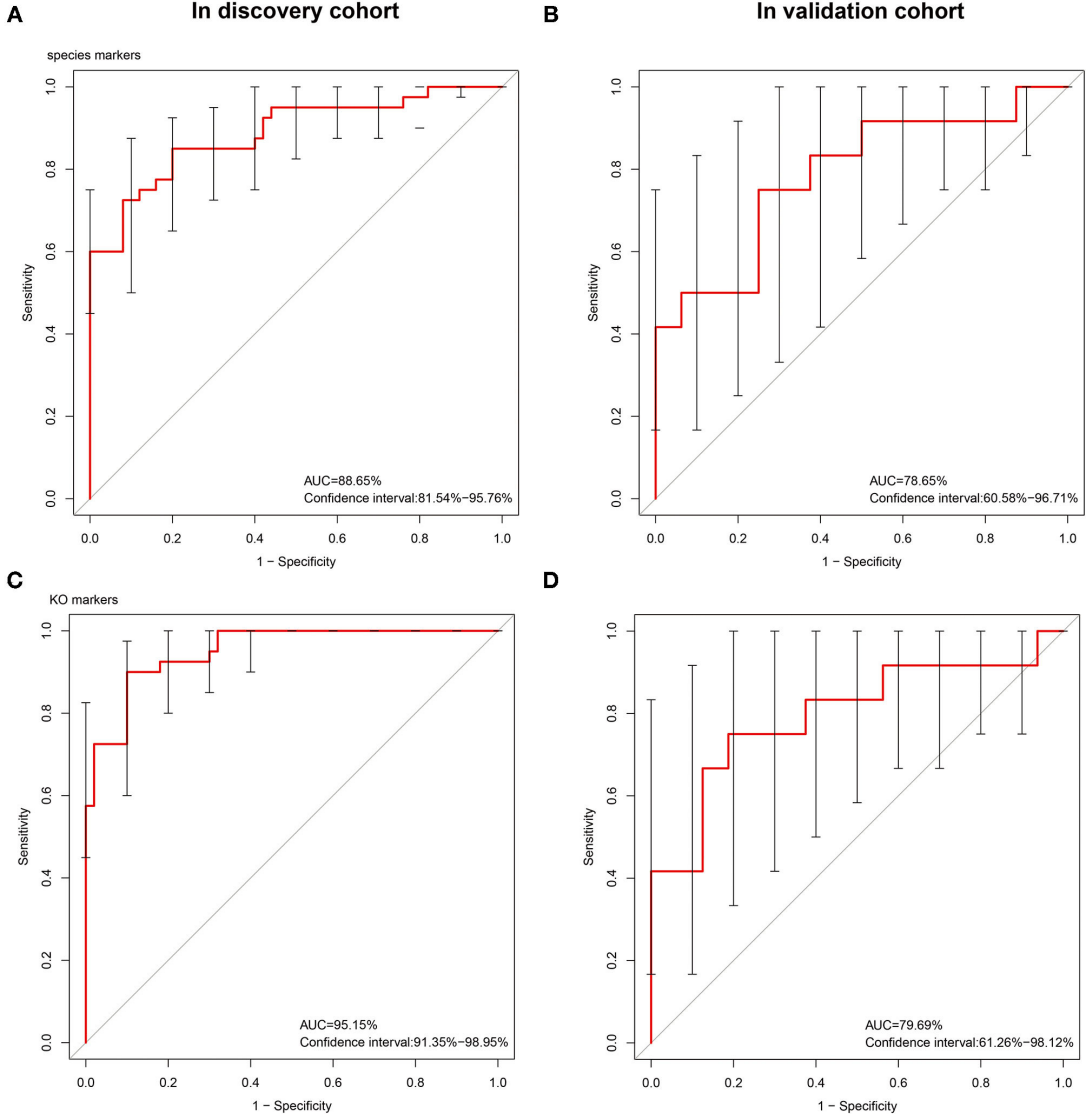
The receiver operating characteristic (ROC) curves of the sequenced reference species markers and the KEEG Ontology (KO) markers. The classification model constructed using 15 species as biomarkers had an AUC of 88.65% in the discovery set **(A)** and 78.65% **(B)** in the validation set. We constructed a classification model using the 10 differentially expressed KO markers with the most advantageous ROC curve values among the 859 differentially expressed KO biomarkers. This model had an AUC of 95.15% **(C)** in the discovery set and 79.69% **(D)** in the verification set.

On the basis of the microbial and gene expression characteristics identified as described above, we next sought to construct a model for distinguishing patients with STC from healthy individuals. First, we selected the 15 species with the most advantageous receiver operating characteristic (ROC) curve values from among the 59 species with differential abundance between the two groups (Table 2). The classification model constructed using these species as biomarkers had an Area Under Curve (AUC) of 88.65% in the discovery set and 78.65% in the validation set (Figure 3). Next, we constructed a classification model using the 10 differentially expressed KO markers with the most advantageous ROC curve values among the 859 differentially expressed KO biomarkers. This model had an AUC of 95.15% in the discovery set and 79.69% in the verification set (Table 3). Thus, the model constructed using KO biomarkers can adequately distinguish between patients with STC and healthy individuals.

**Table 2:** The best 10 KEGG Ontology (KO) markers in the receiver operating characteristic (ROC) curve picked from the 859 differential KO markers.

**KO marker**	**Median (control)**	**Median (STC)**	***P*-value**	**FDR**	**Enrichment in which group**
K00087	0.000007	0.00001	0.0006	0.00004	STC
K00968	0.000006	0.00001	0.0006	0.002	STC
K03205	0.0003	0.0006	0.0006	0.0002	STC
K03696	0.0002	0.0002	0.0006	0.0002	STC
K07138	0.00009	0.0001	0.0006	0.000005	STC
K07444	0.0002	0.0002	0.0006	0.0001	Control
K07650	0.0000006	0.000001	0.004	0.027	STC
K11261	0.000001	0.000008	0.00005	0.00001	STC
K13787	0.000001	0.000003	0.00001	0.0005	STC
K17898	0.0000002	0.000001	0.000000001	0.0001	STC

**Table 3:** The best 15 species markers in the ROC curve picked from the 59 differential species markers.

**Species marker**	**Median (control)**	**Median (STC)**	***P*-value**	**FDR**	**Enrichment in which group**
Akkermansia_muciniphila	0	0.000001	0.0006	0.027	STC
Clostridium_hathewayi	0.000000006	0.000003	0.001	0.001	STC
Clostridium_symbiosum	0	0.000002	0.001	0.03	STC
Coprobacillus_sp_29_1	0	0	0.004	0.09	Control
CoprocoSTCus_catus	0.000002	0.00001	0.007	0.12	STC
Desulfovibrio_desulfuricans	0	0	0.007	0.12	control
Erysipelotrichaceae_bacterium_2_2_44A	0	0.0000001	0.0004	0.027	STC
Fusobacterium_periodonticum	0	0	0.04	0.34	Control
Gordonibacter_pamelaeae	0	0.0000007	0.0005	0.02	STC
Lachnospiraceae_bacterium_3_1_57FAA_CT1	0	0.00000003	0.00008	0.0099	STC
Oscillibacter_sp_KLE_1745	0	0	0.009	0.13	Control
Parabacteroides_merdae	0.00002	0.0001	0.002	0.05	STC
Roseburia_intestinalis	0.00004	0.000003	0.004	0.09	Control
Subdoligranulum_sp_4_3_54A2FAA	0	0	0.001	0.001	Control
Subdoligranulum_unclassified	0.00004	0.0001	0.0001	0.012	STC

## Discussion

In this study, we studied the gut microbiome in patients with STC using quantitative metagenomics. To the best of our knowledge, this is largest metagenomics study performed to date analyzing the characteristics of the gut microbiome in patients with STC. Our results show that the gut microbial characteristics of patients with STC differ from those of healthy controls, suggesting that gut microbiome composition may contribute to the development of STC.

We found that the intestinal flora of patients with constipation is more diverse than that of healthy individuals. In addition, we identified significant differences between the constipated and control groups at the phylum, genus, and species level. There was a statistically significant increase in the abundance of Actinobacteria, Firmicutes, and Verrucomicrobia and a statistically significant decrease in the abundance of Bacteroidetes, Euryarchaeota, Fusobacteria, and Synergistetes in the gut of patients with STC compared with the healthy control group.

However, the abundance of *Roseburia intestinalis*, a prominent butyrate-producing Firmicute that is reported to be the primary degrader of dietary fiber (20), was increased in the healthy group compared with the STC group. This is the first study to robustly demonstrate that *Roseburia intestinalis* abundance is increased in the gut microbiome of patients with STC compared with healthy controls using shotgun sequencing. The altered abundances of this species are a cause or a consequence of constipation that warrant further investigation. In the recent years, the role of *Roseburia intestinalis* has been revealed in more and more studies (21, 22). The administration of *Roseburia intestinalis* contributes to restoration of the gut microbiota, promoting colon repair, and the recovery of gastrointestinal function.

The findings from this study may provide a rationale for microbiome-based therapies and patient stratification in the clinical management of constipation. The molecular Operational Taxonomic Units markers identified the current cohort that need to be validated in larger, independent studies. Although the alterations that we noted in the microbiota could serve as biomarkers for disease diagnosis or severity stratification, animal experiments and *in-vitro* studies are needed to elucidate whether these markers are cause of the disease or simply the consequence of increased CTT leading to stool retention and altered fermentation parameters (23, 24). Further investigation into possible mechanisms, microbial-assisted diagnosis, and therapeutics holds great potential for the effective management of constipation.

We constructed classification algorithms (classifiers) to identify patients with STC using 3 types of biomarkers. Each of these 3 enterotypes are identifiable by the variation in the levels of 1 of 3 genera: Bacteroides (enterotype 1), Prevotella (enterotype 2), and the 3rd enterotypes were marked by Alistipes and Eubacterium (Figure 2; Supplementary Figure S1). These phylogenetic and functional differences among enterotypes, thus, reflect different combinations of microbial trophic chains with a probable impact on synergistic interrelations with the human hosts. The robustness and predictability of the enterotypes in different cohorts and at multiple phylogenetic and functional levels indicate that they are the result of well-balanced, defined microbial community compositions of which only a limited number exist across individuals (25, 26).

The results showed that all the samples clustered into 1 of 3 enterotypes. The Bacteroides and Prevotella enterotypes were identical to those reported earlier in other diseases (27–29) and the 3rd enterotype was distinguished by high levels of Alistipes and Eubacterium, which have never been reported, may be unique to patients with STC, and were different from other types of diseases.

Some strong correlations occur between host properties and particular functions at the gene or module level (a module is a part of a pathway that is functionally tightly interconnected); we also attempted to identify bacterial biomarkers that may help in the diagnosis of STC by generating 10 different metagenomic shotgun markers. When using these biomarkers, we were able to distinguish patients with STC from normal patients with a moderate degree of certainty (AUC = 82.69). This is extremely important for the diagnosis and classification of constipation and serving the clinic through a cost-effective and time-saving technique is one of the purposes of this study.

The large sample size used in this study allowed us to focus on functional metagenomic data that are potentially related to prognosis and could be important for identifying adjuvant therapeutic targets that target functional pathways rather than specific species. Our analysis suggested that there are differences in the fatty acid biosynthesis, fatty acid degradation, butanoate metabolism, and methane metabolism pathways between STC and healthy microbiomes. Thus, modulating these metabolic pathways could influence the development of STC and related symptoms, providing new avenues for exploring the role of the gut microbiome in STC (30).

The most enriched orthologs in patients with STC were related to fatty acid, especially short-chain fatty acids (SCFAs), that play an important role in colonic motility. The intestinal microorganism ferment dietary fiber produces SCFAs, mainly acetic acid, propionic acid, and butyric acid. In the intestine, SCFAs not only act as nutrients for intestinal epithelial cells, but also serve to regulate intestinal pH, cell proliferation and differentiation, and gene expression (31, 32).

We also found that SCFAs have an important role in other diseases. Tan et al. reported low SCFAs in Parkinson's disease were significantly associated with poorer cognition. Lower butyrate levels correlated with worse postural instability-gait disorder scores (33). Liu et al. indicated that altered fecal microbiota might play vital roles in the pathogenesis of pediatric myasthenia gravis by reducing SCFAs. The microbial markers might serve as novel diagnostic methods for pediatric myasthenia gravis (34). Therefore, the intestinal microbiota in patients with STC may affect gut motility by altering host metabolism.

Metagenomics analysis showed that there was significant difference in the methane metabolism pathway between the two groups. This finding is particularly intriguing because one of the main clinical symptoms of patients with constipation is abdominal distension caused by excessive methane production. Compared with the healthy control group, methane metabolism pathways were significantly enriched in the STC group. Previous studies have shown that methane negatively affects intestinal motility, slowing colonic transit time (35, 36). This is the first study to focus on the correlation between methane and STC at the metagenomic level. On the basis of the above findings, we believe that methane quantification could be used to diagnose STC (37, 38).

There are several limitations of this study. First, we have collected for testing is natural feces, which are different from mucosal adherent bacteria. A cause–effect relationship between microbiome and constipation needs to be evaluated, especially for mucosal microbiome. Secondary, a thorough dietary survey was not performed prior to stool collection. Diet is a strong determinant of the microbial microenviroment. Constipated individuals are reported to consume fewer calories as well as lesser amount of protein and fat, but possibly increased amount of fibers and all of the above factors would affect the colonic microbiota and transit time. Thirdly, these diagnosis biomarkers should be tested through a larger clinical sample size and should be discussed to explore the application in future clinical diagnosis and treatment of STC. Fourth, we review the pros and cons of the current high-throughput methods. After isolating whole-genomic DNAs from a fecal sample, the high-throughput identification of 16S ribosomal DNA (rDNA) by Next Generation Sequencing (NGS) alone generates enough information for high-throughput identification of microbes constituting gut microbiota. NGS methods are the best approaches. However, this technology is far from becoming commonplace and affordable because of the high cost and infrastructure required for the analysis. In addition, this method cannot dynamically reflect the changes and effects of the intestinal flora and is gradually being replaced by metabolomics, transcriptomics, proteomics, bacterial culture, and fluorescence in situ hybridization (FISH), which facilitates the understanding the link between individual bacterial cells and their metabolic functions. Our results provide new avenues for the development of novel diagnostic tools and potential treatments. Furthermore, our findings suggest that alteration of the microbiome may lead to constipation by changing the levels of microbial-derived metabolites in the gut and that restoring balance to the disrupted microbiota could help to improve the clinical phenotype. Further experimental or preclinical studies are necessary to examine the role of the altered microbiota in the pathogenesis of colonic motility changes.

## Conclusion

In this study, we have reviewed the association of gut microbiome composition with STC as well as its possible roles in the development of this disease. We revealed a novel link between the gut microbiome, host genome, and pathology of STC. This study will be a platform model of the microbiome studies to elucidate etiology of this disease, evidenced by the changes in genes, pathways, and various taxonomic levels. Discovery of the associated microbes of STC in the gut microbiome may help us to seek more intestinal microbial agents for this disease.

## Data Availability Statement

The data presented in the study are deposited in the: https://www.ncbi.nlm.nih.gov/bioproject/779475 repository, accession number PRJNA779475.

## Ethics Statement

The studies involving human participants were reviewed and approved by the Ethics Committee of Tenth Hospital of Tongji University. The patients/participants provided their written informed consent to participate in this study. Written informed consent was obtained from the individual(s) for the publication of any potentially identifiable images or data included in this article.

## Author Contributions

HT, JC, CW, SZ, and QC conceived the method. ZZ, NQ, XL, and HQ designed the experiments. XL, SZ, CY, JC, and NL performed the experiments and implemented the method. ZZ, CW, HT, NQ, NL, and HQ assessed the data and planned the statistical analyses. HT, BY, JC, CY, and QC wrote and edited the manuscript. All authors contributed to the article and approved the submitted version of the manuscript.

## Funding

This study was supported by the National Natural Science Foundation of China (No. 82100698) and the Clinical Research Plan of Shenkang Hospital Development Center (No. SHDC2020CR4026 and SHDC2020CR1030B).

## Conflict of Interest

ZZ, CW, and NQ was employed by company Realbio Genomics Institute. The remaining authors declare that the research was conducted in the absence of any commercial or financial relationships that could be construed as a potential conflict of interest.

## Publisher's Note

All claims expressed in this article are solely those of the authors and do not necessarily represent those of their affiliated organizations, or those of the publisher, the editors and the reviewers. Any product that may be evaluated in this article, or claim that may be made by its manufacturer, is not guaranteed or endorsed by the publisher.
