# Supplementary material for: Gut Metagenome as a Potential Diagnostic and Predictive Biomarker in Slow Transit Constipation
Source: Front Med (Lausanne). 2022 Feb 8;8:777961. doi: 10.3389/fmed.2021.777961 (PMC8862142; doi:10.3389/fmed.2021.777961)

Additional files 2: Supplementary figures for:

Figure S1. The relative abundance of *Bacteroides*, *Prevotella*, *Alistipes* and *Eubacterium* in STC patient group and healthy control group in discovery cohort.


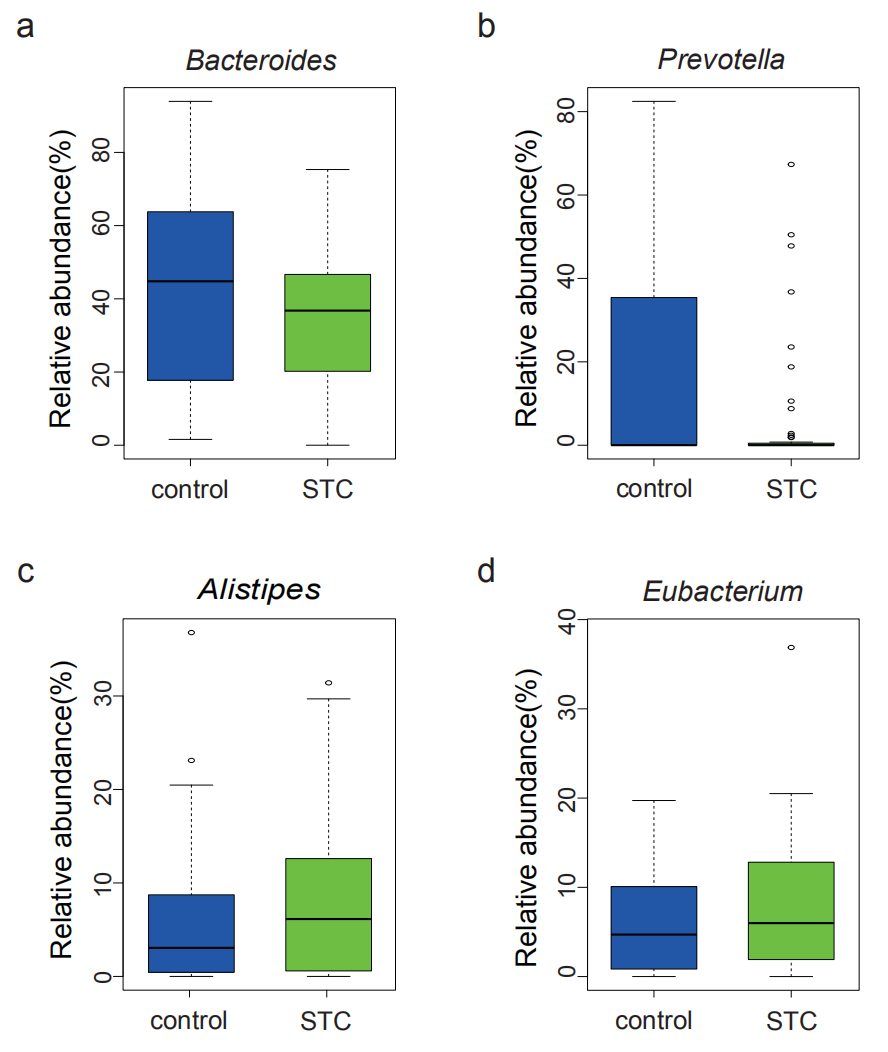


Figure S2. The heatmap of top 30 genus abundance in different enterotypes. Rows are the genus of top 4, based on the average abundance in all samples, the top 4 genera were ranked from high to low by rows. Columns are the samples, based on the abundance of dominant genera in the two enterotypes，samples were ranked from high to low by columns.


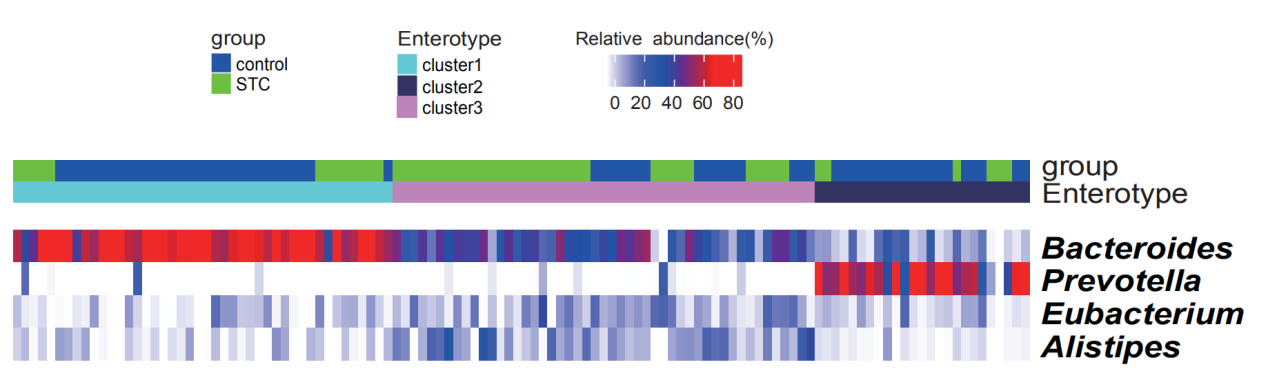


Figure S3. The KO markers in fatty acid biosynthesis pathway. The genes with red frame were enriched in healthy control group and the genes with green frame were enriched in STC patient group.

**
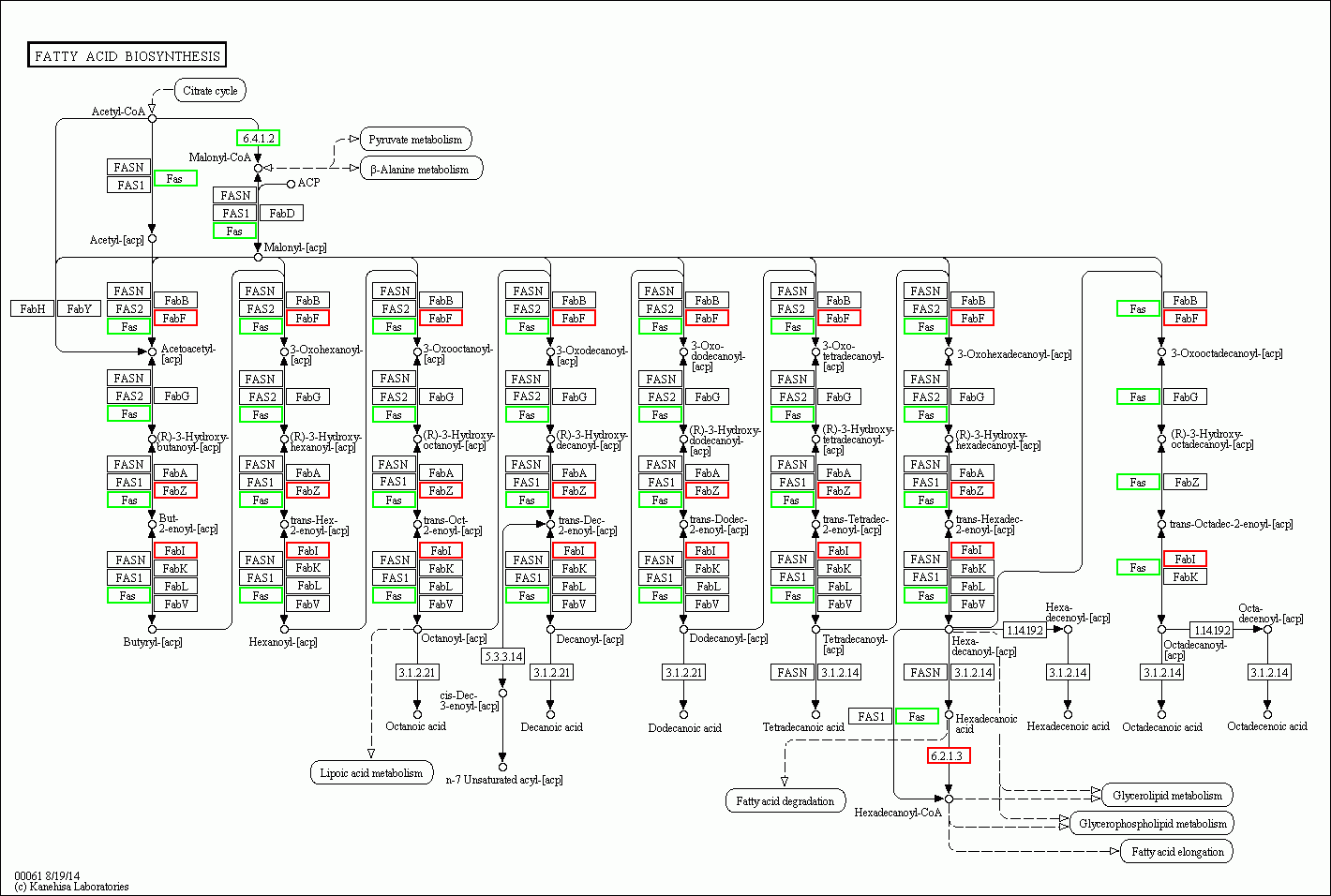
**

Figure S4. The KO markers in fatty acid degradation pathway.The genes with red frame were enriched in healthy control group and the genes with green frame were enriched in STC patient group.

**
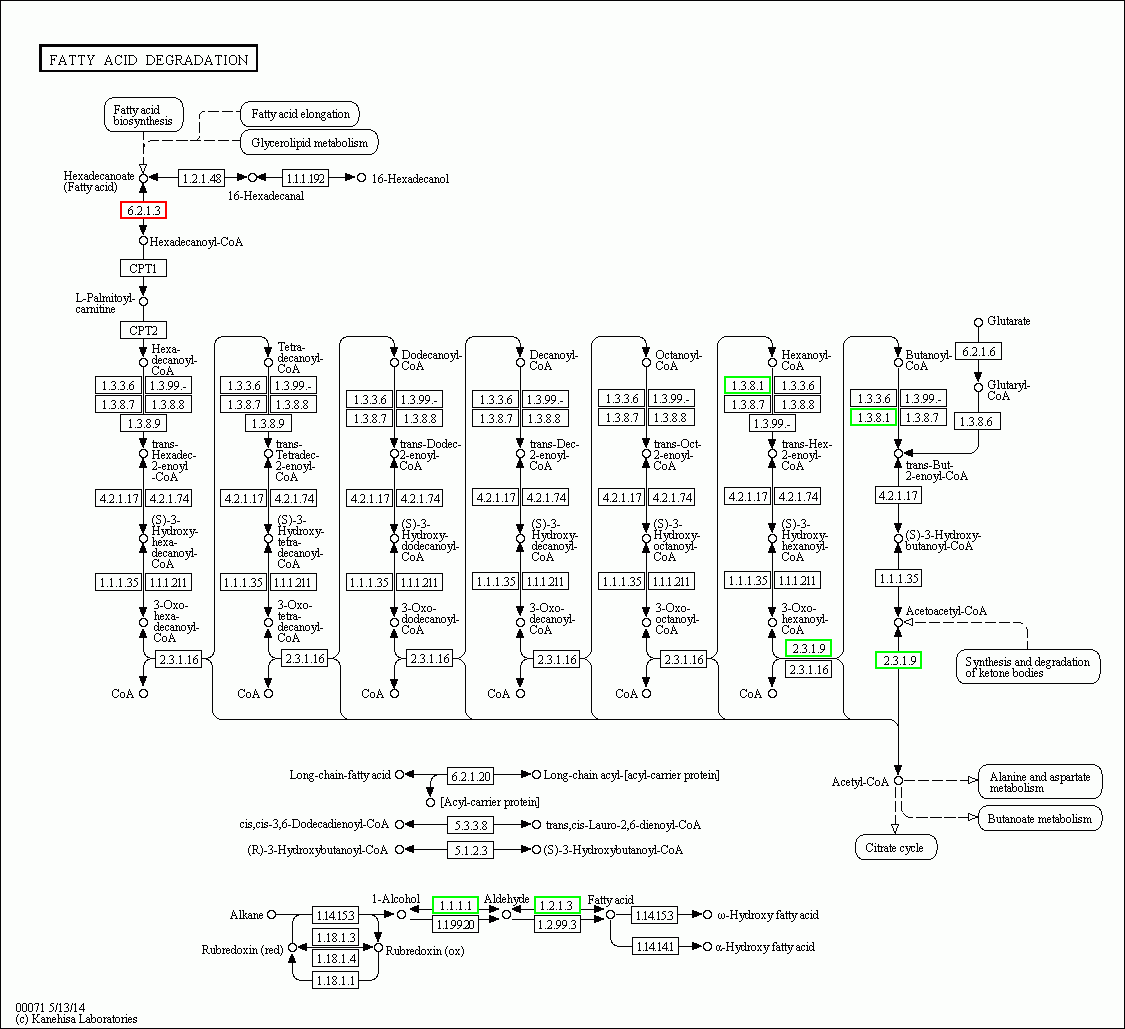
**

Figure S5. The KO markers in butanoate metabolism pathway.The genes with red frame were enriched in healthy control group and the genes with green frame were enriched in STC patient group.


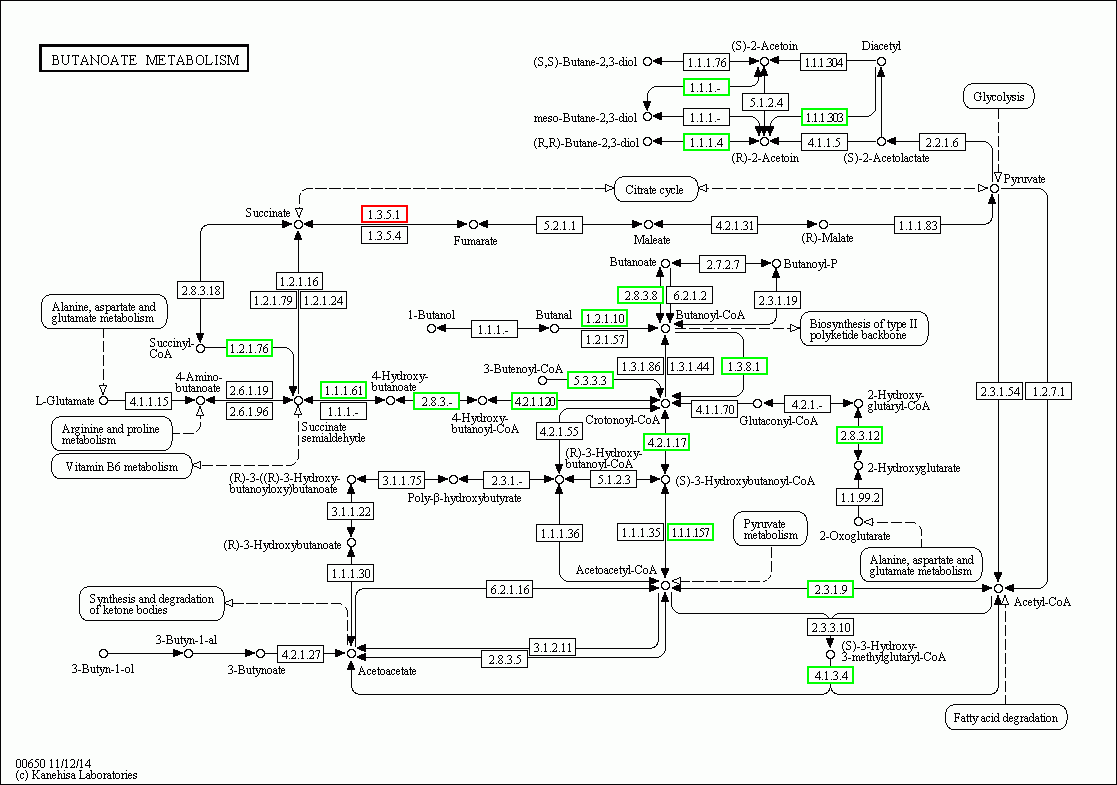


Figure S6. The KO markers in methane metabolism pathway. The genes with red frame were enriched in healthy control group and the genes with green frame were enriched in STC patient group.


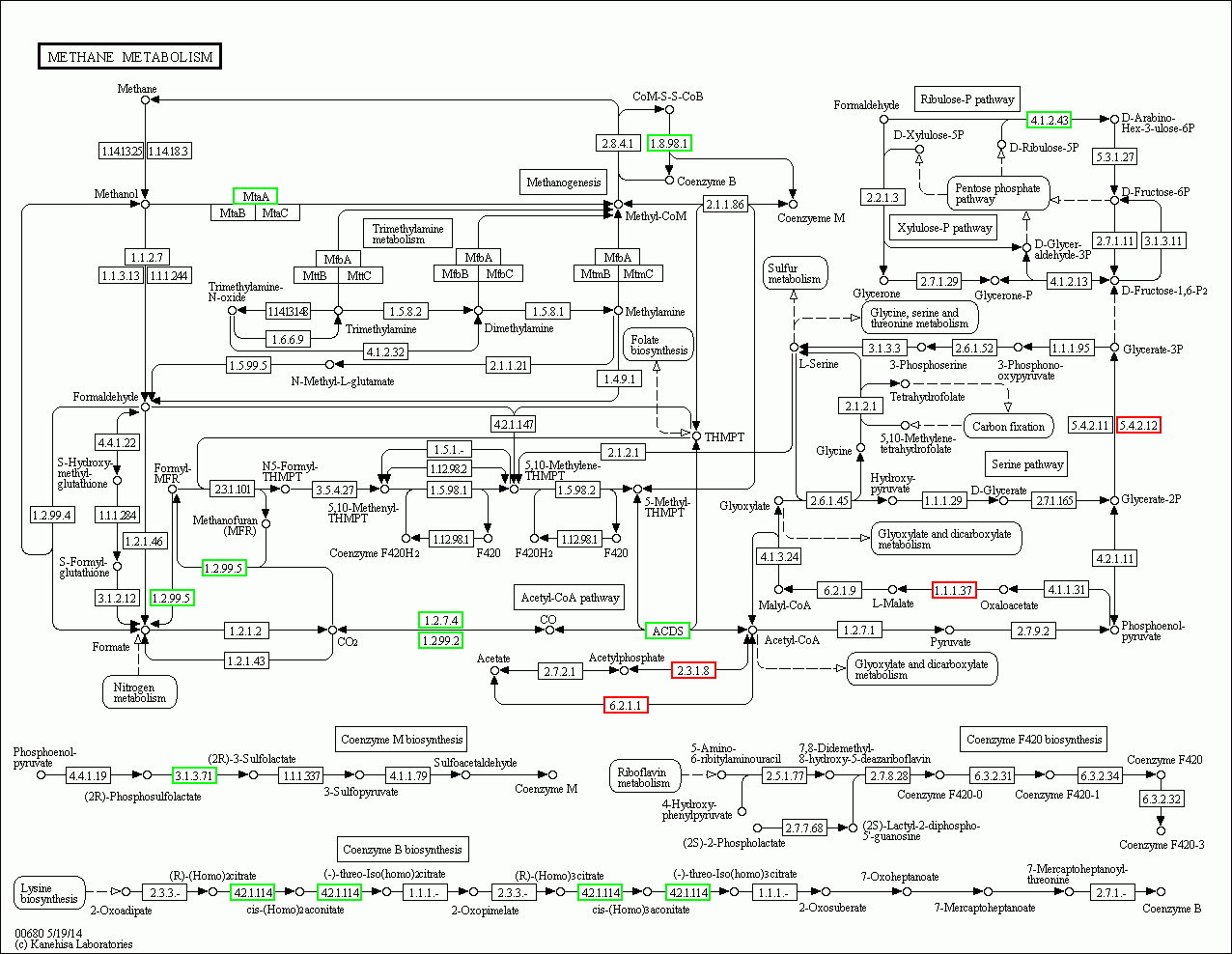

Supplement: Supplementary file 2 [file Data_Sheet_2.docx]
